# Validação do instrumento reduzido Diabetes-21 para avaliação da qualidade de vida relacionada à saúde em pessoas com diabetes

**DOI:** 10.1590/S1679-49742022000100004

**Published:** 2022-03-14

**Authors:** Árlen Almeida Duarte de Sousa, Ana Monique Gomes Brito, Marise Fagundes Silveira, Andréa Maria Eleutério de Barros Lima Martins

**Affiliations:** 1 Universidade Estadual de Montes Claros, Departamento de Métodos e Técnicas Educacionais, Montes Claros, MG, Brasil. Universidade Estadual de Montes Claros Universidade Estadual de Montes Claros Departamento de Métodos e Técnicas Educacionais Montes Claros MG Brazil; 2 Faculdades Unidas do Norte de Minas, Departamento de Enfermagem, Montes Claros, MG, Brasil. Faculdades Unidas do Norte de Minas Faculdades Unidas do Norte de Minas Departamento de Enfermagem Montes Claros MG Brazil; 3 Universidade Estadual de Montes Claros, Programa de Pós-Graduação em Ciências da Saúde, Montes Claros, MG, Brasil. Universidade Estadual de Montes Claros Universidade Estadual de Montes Claros Programa de Pós-Graduação em Ciências da Saúde Montes Claros MG Brazil

**Keywords:** Diabetes *Mellitus*, Qualidade de Vida, Reprodutibilidade dos Testes, Análise Fatorial, Diabetes Mellitus, Calidad de Vida, Reproducibilidad de los Resultados, Análisis Factorial, Diabetes *Mellitus*, Quality of Life, Reproducibility of Results, Factor Analysis, Statistical

## Abstract

**Objetivo:**

Analisar a validade, confiabilidade e interpretabilidade de instrumento reduzido para avaliação da qualidade de vida relacionada à saúde entre pessoas com diabetes *mellitus*.

**Métodos:**

Estudo de validação, composto pelas fases de adaptação do instrumento Diabetes-39 (constituído por 5 dimensões e 39 itens), pré-teste, análises de validade estrutural (exploratória e confirmatória), confiabilidade, validade concorrente e interpretabilidade.

**Resultados:**

A estrutura fatorial da versão final reduzida diferiu do instrumento original. Foram reduzidos os itens, de 39 para 21, e as dimensões, de 5 para 4. As cargas fatoriais, nas análises exploratória e confirmatória, variaram entre 0,41 e 0,90 e entre 0,51 e 0,89, respectivamente. A confiabilidade apresentou-se adequada (alfa de Cronbach=0,91; Kappa≥0,60 em todos os itens; coeficiente de correlação intraclasse=0,91).

**Conclusão:**

O instrumento reduzido Diabetes-21 foi considerado válido, confiável e interpretável para avaliação da qualidade de vida relacionada à saúde entre pessoas com diabetes *mellitus.*

## Resumen

**Objetivo:**

Analizar la validez, confiabilidad y la interpretación del instrumento reducido para evaluar la calidad de vida relacionada con la salud en personas con diabetes mellitus.

**Métodos:**

Estudio de validación compuesto por las fases de: adaptación del instrumento Diabetes-39 (5 dimensiones/39 elementos), pre test, análisis de validez estructural, confiabilidad, validez concurrente y calidad de la interpretación.

**Resultados:**

La estructura factorial de la versión final del instrumento reducido fue diferente a la original. Hubo una reducción de 39 a 21 ítems y de 5 a 4 dimensiones. Las cargas factoriales en los análisis exploratorios y confirmatorios variaron entre 0,41-0,90 y 0,51-0,89, respectivamente. La fiabilidad fue adecuada (alfa de Cronbach=0,91; Kappa≥0,60 en todos los ítems; coeficiente de correlación intraclase=0,91).

**Conclusión:**

El Diabetes-21 se consideró válido, confiable e interpretable para evaluar la calidad de vida relacionada con la salud en personas con diabetes mellitus.

## Abstract (en)

**Objective:**

To analyze the validity, reliability and interpretability of a short form instrument for assessing health-related quality of life among people with diabetes mellitus.

**Methods:**

This was a validation study, comprised of the adaptation phases of the Diabetes-39 instrument (consisting of 5 domains and 39 items), pre-test, structural validity analyses (exploratory and confirmatory), reliability, concurrent validity and interpretability.

**Results:**

The factorial structure of the short final version differed from the original instrument. The items were reduced from 39 to 21 and domains from 5 to 4. The factor loading, in exploratory and confirmatory analyses, ranged between 0.41 and 0.90 and between 0.51 and 0.89, respectively. Reliability was adequate (Cronbach’s alpha=0.91; Kappa≥0.60 in all items; intraclass correlation coefficient =0.91).

**Conclusion:**

Diabetes-21, a short form instrument, was considered valid, reliable and interpretable for assessing health-related quality of life among people with diabetes *mellitus*.

**Contribuições do estudo**

**Principais resultados**

O Diabetes-21 foi considerado válido, confiável e interpretável para avaliar a qualidade de vida relacionada à saúde entre pessoas com diabetes usuárias da atenção primária.

**Implicações para os serviços**

Trata-se de um instrumento com potencial para ser utilizado por profissionais de saúde no âmbito da assistência às pessoas com diabetes, por ser capaz de identificar comprometimento na qualidade de vida e possibilitar a implantação de ações de saúde.

**Perspectivas**

Espera-se que o Diabetes-21 seja utilizado em pesquisas futuras e por serviços de saúde, na identificação de pessoas com necessidades de intervenção, especialmente por demandar menor tempo de aplicação.

## Introdução

O diabetes *mellitus* é uma das principais causas de mortes prematuras e evitáveis no Brasil. A qualidade de vida da população frequentemente afetada por problemas de saúde associados ao diabetes reforça a necessidade de avaliação dessa condição, além do acompanhamento e tratamento clínico da doença.^1^

A qualidade de vida de pessoas com diabetes *mellitus* tem sido avaliada pelo instrumento Diabetes-39, elaborado nos Estados Unidos e adaptado para o Brasil. Trata-se de uma escala multidimensional, composta por 39 itens que avaliam cinco domínios da qualidade de vida da pessoa com diabetes: energia e mobilidade; controle do diabetes; ansiedade e preocupação; sobrecarga social; e funcionamento sexual. Quanto maior o escore obtido na escala, maior o impacto negativo na qualidade de vida das pessoas.^2^^,^^3^

O Diabetes-39 tem sido considerado um instrumento adequado para avaliar a qualidade de vida dessa população. Contudo, em sua adaptação transcultural para a população brasileira, as propriedades psicométricas avaliadas foram apenas a consistência interna e a validade de constructo (validade convergente e discriminante), sendo necessário analisar sua estabilidade, validade estrutural (análises fatoriais exploratória e confirmatória) e interpretabilidade.^3^ Apesar de a versão brasileira manter a validade e confiabilidade da versão original, o instrumento é longo, demanda tempo considerável e ambiente adequado para sua aplicação. Estas limitações do instrumento podem desestimular ou criar barreiras de participação, tanto para pessoas com diabetes quanto para profissionais de saúde, ao passo que questionários curtos apresentam aplicação rápida, são práticos e econômicos.^4^^-^^6^

A verificação da eficácia de instrumentos que avaliam condições de saúde é essencial, pois eles podem apresentar limitações em suas propriedades psicométricas.^7^^-^^9^

Um grupo de pesquisadores especialistas na avaliação de instrumentos de medida desenvolveu o ‘COnsensus-based Standards for the selection of health Measurement INstruments’ (COSMIN), uma *checklist* de análise das propriedades psicométricas dos instrumentos que avaliam condições de saúde. Apresentado em quatro domínios, o COSMIN avalia a validade, confiabilidade, responsividade e interpretabilidade dos instrumentos.^8^^,^^9^ Pesquisas epidemiológicas que utilizam instrumentos testados, quanto a sua validade e confiabilidade, contribuem para as práticas baseadas em evidências na área da saúde.^10^

O Diabetes-39, ao avaliar a qualidade de vida relacionada à saúde de pessoas diabéticas, auxilia na identificação de necessidades de assistência e, consequentemente, na redução do risco de complicações decorrentes da doença. Uma versão reduzida desse instrumento pode contribuir para um menor tempo de sua aplicação em estudos epidemiológicos. Além disso, no processo de validação de um instrumento, obtêm-se cargas fatoriais, ou pesos, que permitem a estimativa de um escore consistente com a importância que cada pergunta/item exerce sobre o construto. Sendo assim, considerar o peso de cada pergunta/item na interpretação do instrumento pode levar a um resultado mais fidedigno.^9^

O estudo teve como objetivo analisar a validade, confiabilidade e interpretabilidade de instrumento reduzido para avaliação da qualidade de vida relacionada à saúde, em pessoas portadoras de diabetes *mellitus*.

## Métodos

### Delineamento

Trata-se de estudo de validação, com etapa transversal, conduzido entre 2016 e 2018, com usuários de unidades da Estratégia Saúde da Família (ESF) no município de Montes Claros, estado de Minas Gerais, Brasil.

### Contexto

O município de Montes Claros, localizado ao norte de Minas Gerais, é o 6º maior do estado em população, ocupa uma área de 3.589,811 km^2^ e apresentava índice de desenvolvimento humano (IDH) de 0,770 em 2010; sua população estimada para 2020 era de 413.487 habitantes.^11^

### População e amostra

A gerência municipal responsável pela ESF foi contatada para a obtenção das listas com a enumeração das unidades-polo das equipes da estratégia. Das 73 unidades-polo existentes em Montes Claros, quatro foram selecionadas mediante sorteio aleatório simples. Dessas quatro, duas foram utilizadas para a avaliação da confiabilidade e validade concorrente, e as duas unidades-polo restantes, para estimar a validade estrutural e interpretabilidade.

Os gestores da ESF das unidades-polo sorteadas forneceram listas com os nomes das pessoas portadoras de diabetes cadastradas e acompanhadas por elas. A partir desses dados, foram consideradas elegíveis para este estudo pessoas com idade maior ou igual a 18 anos, cadastradas na ESF e diagnosticadas com diabetes. Foram excluídas aquelas com três ou mais comorbidades, língua nativa distinta do português, deficiência visual ou auditiva, que apresentavam sinais de intoxicação por álcool ou outras drogas no momento das entrevistas; e os idosos com comprometimento cognitivo, este verificado pelo miniexame do estado mental.^12^ Foram considerados como perdas os instrumentos que apresentaram três ou mais dados incompletos.

Dessa forma, a amostra do estudo foi dividida em dois diferentes grupos:

1. O grupo de confiabilidade (consistência interna e estabilidade) e validade concorrente do instrumento, em que foram selecionadas 50 pessoas com diabetes cadastradas nas unidades, uma vez que amostras constituídas por 50 a 100 pessoas são suficientes para essas etapas;^13^ e
2. O grupo das análises fatoriais exploratória e confirmatória, e de interpretabilidade, em que os participantes foram selecionados a partir do cálculo de uma amostra probabilística para população infinita, considerando-se a proporção de 50% de pessoas com diabetes com comprometimento na qualidade de vida, nível de confiança de 95%, erro amostral de 6 pontos percentuais e acréscimo de 10% para perdas.^14^

### Variáveis

As condições sociodemográficas e econômicas investigadas foram: sexo (masculino; feminino); faixa etária (em anos, categorizada em quartis: 22 a 54; 55 a 61; 62 a 68; 69 ou mais); escolaridade (em anos completos de estudo: 0; 1 a 4; 5 a 8; 9 a 11; 12 ou mais); estado civil (casado/união estável; solteiro/viúvo/divorciado/ separado); raça/cor da pele (branca; amarela; preta; parda; indígena; sem declaração); renda familiar [até R$ 998,00; acima de R$ 998,00 (salário mínimo à época do estudo)]; e gasto com medicamentos (não; sim).

O comprometimento cognitivo, um critério de exclusão do estudo, foi definido de acordo com diferentes pontos de corte - segundo escolaridade - do miniexame do estado mental: 13 para pessoas sem estudo; 18 para baixa e média escolaridade (1 a 8 anos incompletos de estudo); e 26 para alta escolaridade (8 ou mais anos de estudo).^12^

Foram classificados com qualidade de vida afetada (sim; não) os participantes que apresentaram escores abaixo do limite inferior do intervalo de confiança de 95% (IC_95%_) da média em cada dimensão.

### Fontes de dados e mensuração

O instrumento avaliado foi o Diabetes-39, adaptado culturalmente para o idioma português do Brasil.^3^ Seus 39 itens estão distribuídos em cinco domínios: energia e mobilidade; controle do diabetes; carga social; função sexual; ansiedade e preocupação. A versão reduzida do Diabetes-39 foi denominada Diabetes-21. 

Adicionalmente, foi realizada uma alteração na escala de resposta (1 = não foi afetada; 2 = pouco afetada; 3 = às vezes afetada; 4 = muito afetada; 5 = extremamente afetada), em substituição à proposta original do Diabetes-39 (barra horizontal de resposta dividida em caixas, contendo em seu interior os números de 1 a 7).^3^

Para verificar a validade, confiabilidade e interpretabilidade do Diabetes-21, as seguintes etapas foram aplicadas: pré-teste, validade estrutural (análise fatorial exploratória e confirmatória), validade concorrente, confiabilidade (consistência interna e estabilidade) e interpretabilidade.

Para o pré-teste, foram entrevistados 20 indivíduos com diabetes cadastrados na primeira unidade sorteada para compor o estudo, selecionados por conveniência. O objetivo dessa etapa foi analisar a aplicabilidade do instrumento antes de sua utilização na amostra final do estudo. Após o pré-teste, os entrevistadores foram convidados a realizar um encontro com os pesquisadores, para relatar sua percepção em relação à aplicação do instrumento com nova escala de resposta. Esse encontro foi conduzido como grupo focal, composto por dez juízes que atuavam como pesquisadores e/ou na assistência à saúde de pessoas com diabetes (2 endocrinologistas, 2 epidemiologistas, 2 enfermeiros, 1 fisioterapeuta, 1 nutricionista, 1 educadora física e 1 cirurgião-dentista).

Além do Diabetes-39, o estudo utilizou o instrumento proposto pela Organização Mundial da Saúde (OMS) para avaliar a qualidade de vida, em sua versão abreviada: World Health Organization Quality Of Life-bref (WHOQOL-bref). Trata-se de um instrumento para avaliação da qualidade de vida, não específico para pessoas com diabetes, composto de duas perguntas de aspecto geral e 26 relacionadas a quatro domínios: físico, psicológico, ambiental e social. Suas respostas consideram uma escala Likert que afere intensidade, frequência, capacidade e avaliação. Os escores de cada domínio foram transformados em uma escala de 0 a 100 invertida e dicotomizada pelo limite inferior do IC_95%_ da média. Indivíduos que alcançaram escores inferiores a esse limite, em cada dimensão, foram classificados com sua qualidade de vida afetada.^15^

### Controle de viés

Os participantes - de todas as etapas do estudo - foram entrevistados em sua própria residência, de forma individual e em ambiente reservado. 

O viés de aferição foi minimizado, por meio do treinamento teórico e prático dos entrevistadores. Estes foram capacitados no sentido de minimizar a subjetividade intrínseca das entrevistas. O treinamento contou com pessoas com diabetes que não participaram do estudo.

### Análises estatísticas

Realizou-se a análise descritiva das variáveis categóricas, estimando-se as frequências absolutas e relativas. Para as variáveis contínuas, foram calculadas a média e o desvio-padrão (DP), e estimados os IC_95%_, além de valores mínimos e máximos.

Com o propósito de estimar a validade concorrente, a correlação de Spearman foi utilizada para verificar a associação entre as variáveis dos instrumentos (WHOQOL-bref e Diabetes-21; Diabetes-39 e Diabetes-21), uma vez que os dados não apresentaram distribuição normal. Em seguida, aplicou-se o teste de correlação entre as escalas geradas por esses mesmos instrumentos. As correlações dos escores do Diabetes-21 com a idade e com a escolaridade dos participantes também foram analisadas.

A confiabilidade do instrumento foi mensurada por meio da consistência interna e da estabilidade. A consistência interna foi testada pelo cálculo do alfa de Cronbach (α), sendo considerados aceitáveis valores ≥0,7.^13^ Para estimar a estabilidade do instrumento, foi aplicado o teste-reteste, em que se verificou a capacidade do instrumento de produzir resultados idênticos, medindo o evento nos mesmos participantes em situações diferentes, por meio do cálculo do coeficiente de correlação intraclasse. O teste-reteste foi aplicado em um intervalo de 7 a 14 dias.^5^^,^^13^ O coeficiente Kappa ponderado foi calculado para avaliar a concordância de cada um dos itens do instrumento reduzido, considerando-se como aceitável o ponto de corte ≥0,60.^16^

A avaliação da validade estrutural do Diabetes-39 foi feita por meio das análises fatoriais exploratória e confirmatória:

- Na análise exploratória, realizou-se a análise da matriz de correlação das variáveis do instrumento (considerou-se significativo p-valor<0,05), seguida dos testes e análises:^17^^-^^19^ (i) o teste de Kaiser-Meyer-Olkin (variando de 0 a 1; valores <0,50 indicam inadequação do método);^18^ (ii) o teste de esfericidade de Bartlett (p-valor<0,05 indica que a matriz de correlação difere de uma matriz-identidade e, portanto, há relacionamentos entre as variáveis incluídas na análise); (iii) a identificação das comunalidades (estimativa da variância compartilhada, ou em comum, entre variáveis) e sua contribuição para cada item (>0,5, níveis de explicação aceitáveis; valores menores ou iguais indicam que o item deve ser excluído); (iv) a análise das cargas fatoriais de cada item em relação aos componentes extraídos (0,40 como limite aceitável da contribuição do item na criação do fator); (v) a definição do número de fatores, baseada na avaliação gráfica do *scree plot* (gráfico dos autovalores *versus* número de fatores por ordem de extração), na verificação do autovalor (superior a 1) e na observação da percentagem de variância total acumulada; e (vi) a análise dos componentes principais, em que as variáveis foram rotacionadas - rotação ortogonal varimax.
- Na análise fatorial confirmatória, aplicou-se o método da máxima verossimilhança.^18^ Para ajustar o modelo, foram considerados os seguintes índices:^19^^,^^20^ (i) a razão entre o qui-quadrado de Pearson e os graus de liberdade (excelente = 1-2; bom = 2-3; aceitável = 4-5; rejeitado = >5); (ii) o índice de qualidade do ajuste (valores adequados: ≥0,90); (iii) o erro quadrático médio da raiz da aproximação (adequados <0,08); (iv) o índice de ajuste comparativo (valores adequados: ≥0,90); e (v) o índice de Tucker-Lewis (valores adequados: ≥0,90).

A sequência das questões foi reorganizada dentro do instrumento reduzido. Para interpretá-lo, optou-se por aplicar o método aditivo ponderado, considerando-se a carga fatorial atribuída a cada um dos itens; por exemplo: Questão 9 x 0,63 (carga fatorial).^13^ Essas estimativas foram feitas por meio da razão entre a soma dos distintos itens que constituem os fatores, multiplicados pelos respectivos pesos fatoriais (carga fatorial), e a soma dos pesos fatoriais atribuídos, sendo considerada a melhor qualidade de vida aquela que consegue o menor escore, uma vez que se trata de uma escala de resposta de tipo Likert, de 1 (não foi afetada) a 5 (extremamente afetada). A variável foi transformada em variável categórica binária ao se assumir um ponto de corte; nesse sentido, os valores foram transformados em uma escala de 0 a 100^14^^,^^21^^,^^22^ invertida e dicotomizada pelo limite inferior do IC_95%_ da média. Assim, os participantes que apresentaram escores inferiores a esse limite em cada dimensão (energia e mobilidade, 35,91; controle do diabetes e carga social, 32,49; função sexual, 31,47; ansiedade e preocupação, 38,10) foram considerados com qualidade de vida afetada.^22^

Em todas as análises, foram utilizados os programas Statistical Package for the Social Science (SPSS) versão 24.0 e Microsoft Excel.

### Aspectos éticos

O projeto do estudo foi aprovado pelo Comitê de Ética em Pesquisa da Universidade Estadual de Montes Claros (CEP/Unimontes) em 22 de março de 2016: Parecer nº 1.461.818; Certificado de Apresentação para Apreciação Ética (CAAE) nº 54417616.1.0000.5146. Todos os participantes assinaram o Termo de Consentimento Livre e Esclarecido.

## Resultados

Os 50 participantes da etapa de verificação da confiabilidade e validade concorrente apresentaram média de idade de 61 anos (DP=11,1; valor mínimo, 36; valor máximo, 87) e escolaridade média de 7,5 anos (DP=3,9; valor mínimo, 0; valor máximo, 12). Nesta etapa, não houve exclusões, perdas ou recusas dos participantes.

**Figura 1:**
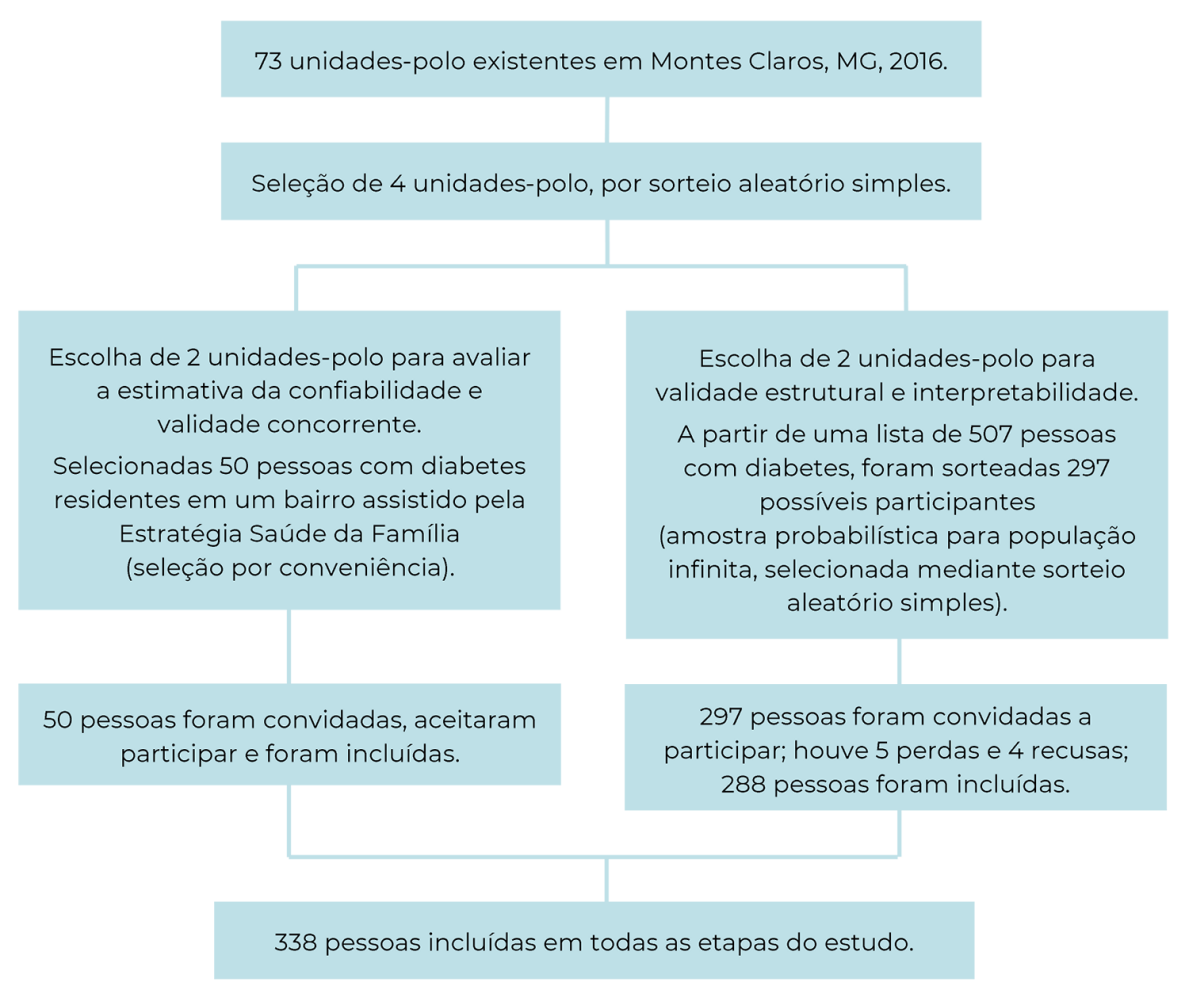
Processo de seleção de pessoas com diabetes *mellitus* assistidas pela Estratégia Saúde da Família, Montes Claros, Minas Gerais, 2019

Foram convidados 297 participantes para a etapa de análise estrutural e interpretabilidade (Figura 1). Entretanto, devido a 5 perdas e 4 recusas, 288 pessoas com diabetes participaram do estudo, com média de idade de 60 anos (variação de 22 a 92 anos: DP=11,7). As informações sociodemográficas, econômicas e sobre a qualidade de vida dos participantes encontram-se na Tabela 1.

**Tabela 1:** Distribuição das características sociodemográficas e econômicas e qualidade de vida, entre pessoas com diabetes *mellitus* (n=338) assistidas pela Estratégia Saúde da Família, Montes Claros, Minas Gerais, 2019

Condições sociodemográficas e econômicas	Populações de estudo			Qualidade de vida afetada nas dimensões do Diabetes-21 (n=288)															
	n=50	n=288		Função sexual				Energia e mobilidade				Controle do diabetes e carga social				Ansiedade e preocupação			
	Total			Sim		Não		Sim		Não		Sim		Não		Sim		Não	
	N	N	%	N	%	N	%	N	%	N	%	N	%	N	%	N	%	N	%
**Sexo**																			
Feminino	34	189	65,6	133	73,1	56	52,8	93	59,2	96	73,3	100	64,1	89	67,4	87	60	102	71,3
Masculino	16	99	34,4	49	26,9	50	47,2	64	40,8	35	26,7	56	35,9	43	32,6	58	40	41	28,7
**Faixa etária (anos)^a^**																			
22-54	8	81	28,1	50	27,5	31	29,3	39	24,8	42	32,1	37	23,7	44	33,4	29	20	52	36,3
55-61	14	62	21,5	39	21,4	23	21,7	35	22,3	27	20,6	30	19,2	32	24,2	28	19,3	34	23,8
62-68	11	78	27,1	43	23,6	35	33	45	28,7	33	25,2	46	29,5	32	24,2	45	31	33	23,1
≥69	11	67	23,3	50	27,5	17	16	38	24,2	29	22,1	43	27,6	24	18,2	43	29,7	24	16,8
**Escolaridade (anos)^a^**																			
0	2	17	6	12	6,7	5	4,7	8	5,3	9	7	8	5,1	9	6,8	8	5,5	9	6,2
01-04	11	65	22,6	37	20,3	28	26,7	28	17,9	37	28,2	34	21,9	31	23,5	30	20,8	35	24,5
05-08	11	89	31	59	32,4	30	28,6	52	33,3	37	28,2	48	31	41	31,1	43	29,9	46	32,2
09-11	10	49	17,1	31	17	18	17,1	28	17,9	21	16	28	18,1	21	15,9	25	17,4	24	16,8
≥12	8	67	23,3	43	23,6	24	22,9	40	25,6	27	20,6	37	23,9	30	22,7	38	26,4	29	20,3
**Estado civil**																			
Casado/união estável	33	174	60,4	103	56,5	71	67	96	61,1	78	59,5	88	56,4	86	65,2	85	58,6	89	62,2
Solteiro/viúvo/divorciado/separado	17	114	39,6	79	43,5	35	33	61	38,9	53	40,5	68	43,6	46	34,8	60	41,4	54	37,8
**Raça/cor da pele**																			
Branca	22	89	30,9	58	31,9	31	29,2	47	29,9	42	32,1	46	29,5	43	32,5	46	31,7	43	30
Amarela	4	14	4,9	7	3,8	7	6,6	5	3,2	9	6,9	5	3,2	9	6,8	5	3,4	9	6,3
Preta	4	36	12,5	25	13,7	11	10,4	20	12,7	16	12,2	16	10,3	20	15,2	14	9,7	22	15,4
Parda	20	134	46,5	82	45,1	52	49,1	75	47,8	59	45	79	50,6	55	41,7	72	49,7	62	43,4
Indígena	-	3	1	2	1,1	1	0,9	3	1,9	-	0	3	1,9	-	0	2	1,4	1	0,7
Sem declaração	-	12	4,2	8	4,4	4	3,8	7	4,5	5	3,8	7	4,5	5	3,8	6	4,1	6	4,2
**Renda familiar^a^**																			
Até R$ 998,00	8	81	30,2	54	31,8	27	27,6	38	26,2	43	35	38	26,2	43	35	34	25,6	47	34,8
Acima de R$ 998,00	42	187	69,8	116	68,2	71	72,4	107	73,8	80	65	107	73,8	80	65	99	74,4	88	65,2
**Gasto com medicamento**																			
Sim	-	147	51	89	48,9	52	49,1	79	50,3	62	47,3	78	50	63	47,7	74	51	67	46,9
Não	-	141	49	93	51,1	54	50,9	78	49,7	69	52,7	78	50	69	52,3	71	49	76	53,1

a) Quantidade de respondentes inferior ao número de participantes, devido a recusa em responder a essa variável.

A consistência interna do Diabetes-21 foi elevada (α=0,91). Além disso, a retirada de qualquer um dos itens não alterou a consistência interna de seu constructo (todos os itens apresentaram α=0,91). A concordância dos 21 itens do instrumento foi adequada (Kappa ≥0,60) (Tabela 2). A estabilidade foi boa (coeficiente de correlação intraclasse=0,91).

**Tabela 2:** Concordância dos domínios, a partir da retirada de cada item do Diabetes-21 (n=50), entre pessoas com diabetes *mellitus* assistidas pela Estratégia Saúde da Família, Montes Claros, Minas Gerais, 2019

Item e domínios	Kappa ponderado	p-valor^a^
**Energia e mobilidade**		
1. Pela sensação de fraqueza	0,67	<0,001
2. Pelo quanto você consegue andar	0,60	<0,001
3. Pela necessidade de realizar exercícios regularmente	0,85	<0,001
4. Por não ser capaz de fazer atividades domésticas ou outros trabalhos que estão relacionados com a casa	0,93	<0,001
5. Pela necessidade de descansar várias vezes no dia	0,83	<0,001
6. Por dificuldades em cuidar de você mesmo(a) (de se vestir, tomar banho ou usar o vaso sanitário)	0,76	<0,001
7. Por andar mais devagar que os outros	0,92	<0,001
**Controle do diabetes e carga social**		
8. Pelas restrições alimentares necessárias para o controle do seu diabetes	0,82	<0,001
9. Por perder o controle dos níveis de açúcar no sangue	0,76	<0,001
10. Por ter que testar os seus níveis de açúcar	0,77	<0,001
11. Por tentar manter seu diabetes bem controlado	0,82	<0,001
12. Pela necessidade de comer em intervalos regulares	0,83	<0,001
13. Pelas restrições que seu diabetes impõe sobre sua família e amigos	0,84	<0,001
14. Pelo constrangimento por ter diabetes	0,73	<0,001
15. Por fazer coisas que sua família ou seus amigos não fazem	0,87	<0,001
**Função sexual**		
16. Pelo diabetes interferir na sua vida sexual	0,61	<0,001
17. Por problemas com função sexual	0,90	<0,001
18. Pela diminuição do interesse pelo sexo	0,87	<0,001
Ansiedade e preocupação1		
19. Pela preocupação relacionada com questões financeiras	0,82	<0,001
20. Pelo estresse ou pressão em sua vida	0,90	<0,001
21. Por sentimento de tristeza ou depressão	0,90	<0,001

a) Teste Z, Kappa ponderado.

A estrutura fatorial da versão final do Diabetes-21 apresentou diferenças em relação à estrutura original. As questões de números 1, 3, 4, 6, 7, 12, 13, 14, 16, 18, 25, 27, 31, 33, 35, 37, 38 e 39 do Diabetes-39 foram retiradas, pois apresentaram comunalidade inferior a 0,5. Assim ela se reduziu, de 39 para 21 itens, com diminuição do número de dimensões de 5 para 4, observada na análise fatorial exploratória (energia e mobilidade; controle do diabetes e carga social; função sexual; ansiedade e preocupação), por conta da união das dimensões ‘controle do diabetes’ e ‘carga social’. A união dessas duas dimensões resultou em melhor consistência interna e estabilidade do instrumento. Todas as dimensões apresentaram boa consistência interna (α ≥0,70). As cargas fatoriais variaram de 0,41 (item 15) a 0,90 (item 17); a dimensão ‘função sexual’ apresentou maior carga fatorial (valores >0,75) (Tabela 3).

**Tabela 3:** Comunalidades, cargas fatoriais de cada item por fator extraído e alfa de Cronbach (α) por fator do Diabetes-21 (n=288),^a^ entre pessoas com diabetes *mellitus* assistidas pela Estratégia Saúde da Família, Montes Claros, Minas Gerais, 2019

Diabetes-21 Item e domínio	Comunalidades	Fator 1	Fator 2	Fator 3	Fator 4	Alfa de Cronbach
**Energia e mobilidade**						
1. Pela sensação de fraqueza	0,47	0,49	0,20	0,16	0,41	0,83
2. Pelo quanto você consegue andar	0,62	0,72	0,26	0,03	0,19	
3. Pela necessidade de realizar exercícios regularmente	0,46	0,59	0,34	-0,01	0,01	
4. Por não ser capaz de fazer atividades domésticas ou outros trabalhos que estão relacionados com a casa	0,61	0,74	0,07	0,21	0,10	
5. Pela necessidade de descansar várias vezes no dia	0,57	0,70	0,18	0,17	0,17	
6. Por dificuldades em cuidar de você mesmo(a) (de se vestir, tomar banho ou usar o vaso sanitário)	0,43	0,61	0,05	0,20	0,11	
7. Por andar mais devagar que os outros	0,61	0,67	0,298	0,12	0,25	
**Controle do diabetes e carga social**						
8. Pelas restrições alimentares necessárias para o controle do seu diabetes	0,55	0,06	0,73	-0,07	0,11	0,83
9. Por perder o controle dos níveis de açúcar no sangue	0,46	0,28	0,54	0,22	0,20	
10. Por ter que testar os seus níveis de açúcar	0,60	0,24	0,71	0,06	0,17	
11. Por tentar manter seu diabetes bem controlado	0,63	0,17	0,73	0,23	0,13	
12. Pela necessidade de comer em intervalos regulares	0,58	0,28	0,66	0,23	0,13	
13. Pelas restrições que seu diabetes impõe sobre sua família e amigos	0,62	0,33	0,64	0,23	0,22	
14. Pelo constrangimento por ter diabetes	0,49	0,14	0,47	0,29	0,40	
15. Por fazer coisas que sua família ou seus amigos não fazem	0,54	0,57	0,41	0,10	0,19	
**Função sexua**l						
16. Pelo diabetes interferir na sua vida sexual	0,79	0,16	0,14	0,85	0,10	0,86
17. Por problemas com função sexual	0,84	0,09	0,14	0,90	0,07	
18. Pela diminuição do interesse pelo sexo	0,71	0,29	0,20	0,76	0,11	
**Ansiedade e preocupação**						
19. Pela preocupação relacionada com questões financeiras	0,56	0,15	0,16	0,07	0,71	0,70
20. Pelo estresse ou pressão em sua vida	0,77	0,16	0,19	-0,00	0,84	
21. Por sentimento de tristeza ou depressão	0,55	0,32	0,22	0,23	0,59	

a) Análise realizada a partir da extração dos fatores pelo método das componentes principais, seguida de uma rotação ortogonal varimax.

Os autovalores encontrados em cada fator foram: 1,48 (energia e mobilidade, fator 1); 1,18 (controle do diabetes e carga social, fator 2); 8,00 (função sexual, fator 3); e 1,77 (ansiedade e preocupação, fator 4). A variância, explicada para cada fator, foi de 7,1% (fator 1), 5,6% (fator 2), 38,1% (fator 3) e 8,5% (fator 4). 

Os resultados do teste de Kaiser-Meyer-Olkin (0,91) e do teste de esfericidade de Bartlett (2.579,51) do Diabetes-21 foram satisfatórios e apropriados (p<0,001). Sua estrutura foi explicada por quatro fatores latentes, indicada pela avaliação gráfica do *scree plot* (ponto de inflexão da curva). Os fatores explicaram 59,3% da variância total. A versão final do Diabetes-21 encontra-se como material suplementar (Material Suplementar 1).

**Figura 2:**
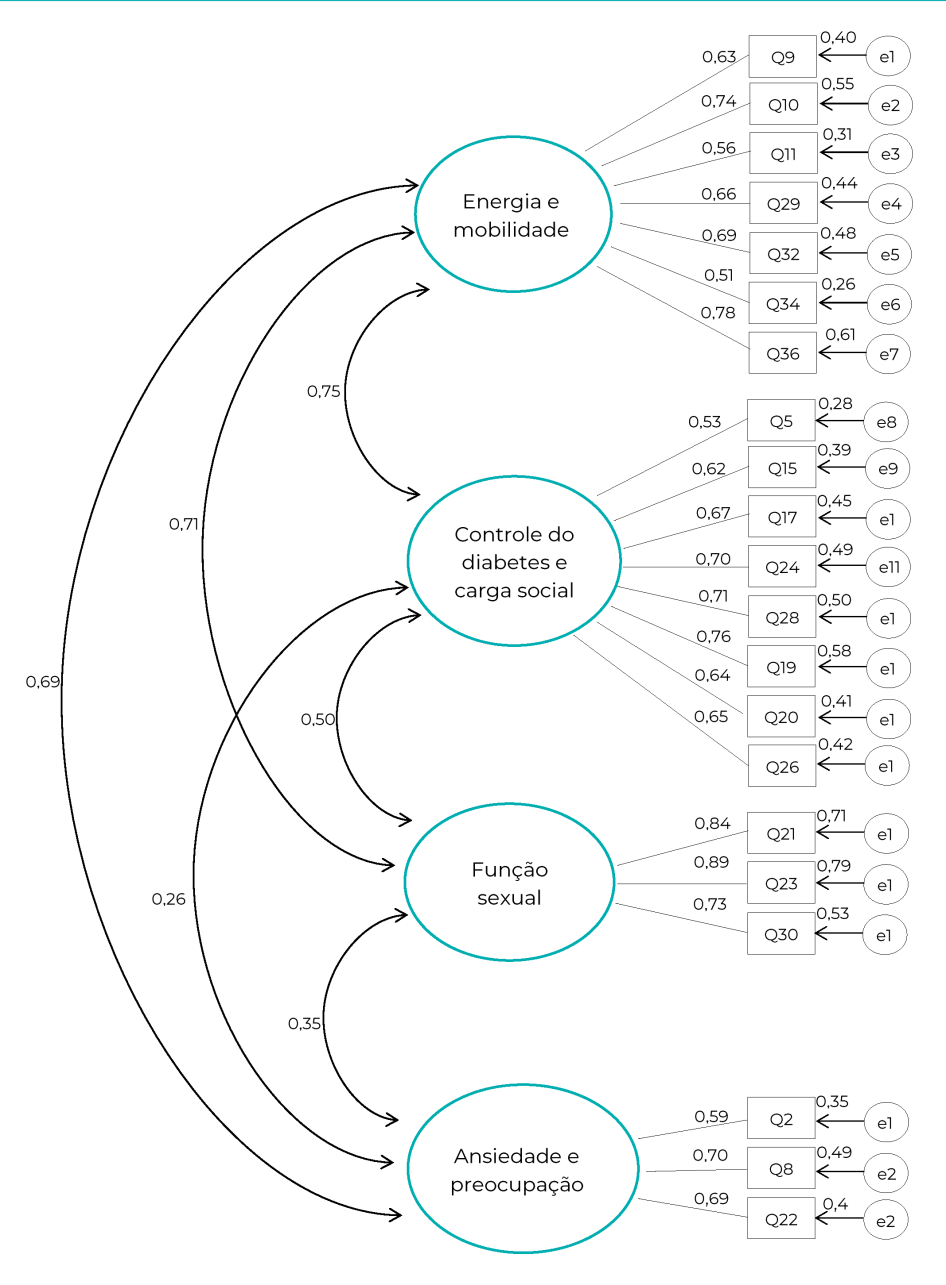
Análise fatorial confirmatória do Diabetes-21 (n=288) entre pessoas com diabetes *mellitus* assistidas pela Estratégia Saúde da Família, Montes Claros, Minas Gerais, 2019

A análise fatorial confirmatória revelou índices adequados ao modelo testado (razão entre o qui-quadrado e os graus de liberdade, 2,22; índice de qualidade do ajuste, 0,88; erro quadrático médio da raiz da aproximação, 0,06; índice de ajuste comparativo, 0,91; índice de Tucker-Lewis, 0,90). As cargas fatoriais variaram de 0,51 (Q34) a 0,89 (Q23). A Figura 2 sistematiza a análise fatorial confirmatória do Diabetes-21.

Houve correlação negativa com a idade (correlação de Spearman = -0,14; p=0,021) e positiva com a escolaridade (correlação de Spearman = 0,76; p=0,001). O valor do coeficiente de correlação de Spearman entre o escore total do Diabetes-39 e o Diabetes-21 foi forte (0,97; p<0,001).

Os valores médios encontrados nas dimensões do Diabetes-21 foram: energia e mobilidade (37,85; DP=16,71; IC_95%_ 35,91;39,78); controle do diabetes e carga social (34,22; DP=14,93; IC_95%_ 32,49;35,96); função sexual (33,77; DP=19,80; IC_95%_ 31,47;36,06); ansiedade e preocupação (40,03; DP=16,60; IC_95%_ 38,10;41,95).

A baixa qualidade de vida relacionada à saúde foi mais frequente no domínio ‘função sexual’ (63,2%), seguido dos domínios ‘energia e mobilidade’ (54,5%; n=157), ‘controle do diabetes e carga social’ (54,2%; n=156) e ‘ansiedade e preocupação’ (50,3%; n=145).

## Discussão

O instrumento Diabetes-21 mostrou-se válido, confiável e interpretável pela população usuária da atenção primária à saúde em Montes Claros, Minas Gerais. Na análise fatorial exploratória, identificou-se associação entre os itens presentes nas dimensões ‘carga social’ e ‘controle do diabetes’, e a união dessas dimensões foi realizada. A análise fatorial confirmatória apresentou ajustes satisfatórios para a aceitação do modelo teórico proposto. Quanto à validade concorrente do Diabetes-21, constatou-se que os maiores níveis de qualidade de vida foram correlacionados positivamente com a escolaridade; e negativamente com a idade. As estimativas da confiabilidade, geral e dentro de cada dimensão, evidenciaram uma confiabilidade satisfatória, tanto na estabilidade quanto na consistência interna. O Diabetes-21 foi capaz de discriminar níveis de qualidade de vida entre os participantes, apresentando-se como uma ferramenta de boa confiabilidade e validade.

Algumas limitações do estudo devem ser consideradas. Utilizou-se amostragem por conveniência para verificação da confiabilidade e validade concorrente; esse tipo de amostragem apresenta limitações, em relação aos resultados e conclusões, uma vez que não podem ser generalizados - com confiança - para uma população total, devido ao viés de seleção. Entretanto, os resultados dessa amostra evidenciam uma variabilidade quanto às variáveis investigadas, sugerindo que ela pode representar a população total. A validade de critério contempla as estimativas das validades concorrente e preditiva, enquanto a validade preditiva não pôde ser estimada por inexistência de padrão ouro.^9^ Sendo assim, foi estimada a validade concorrente, por meio da correlação entre os escores obtidos do Diabetes-21 com a idade e a escolaridade dos participantes. A responsividade não foi estimada por se tratar de estudo transversal, não idealizado para detectar mudanças ao longo do tempo.

Não foram encontrados estudos de validação estrutural do Diabetes-39 com redução de itens ou dimensões. Sua redução, no presente estudo, baseou-se na necessidade de revisar instrumentos antes de sua aplicação em amostras específicas, porque tanto os fatores intrínsecos como os extrínsecos à doença podem sofrer modificações no decorrer do tempo. Os resultados encontrados nesta investigação corroboram aqueles registrados em estudos prévios, pois, ao se comparar o instrumento Diabetes-39 com outros instrumentos, ele foi considerado adequado, sendo previamente utilizado na investigação da qualidade de vida entre pessoas com diabetes.^23^^-^^25^

No Diabetes-21 observou-se que a baixa qualidade de vida foi mais frequente na dimensão ‘função sexual’. Diversos fatores relacionam-se a disfunção sexual entre pessoas com diabetes. As anormalidades vasculares e endócrinas, o impacto emocional e os medicamentos utilizados no tratamento podem gerar problemas de ejaculação, disfunção erétil e na excitação, e assim, diminuição do desejo.^26^^,^^27^ Nesse sentido, a função sexual pode ser considerada no tratamento do diabetes, da mesma forma que quaisquer aspectos emocionais podem impactar na qualidade de vida das pessoas.^23^

O valor médio encontrado no domínio ‘controle do diabetes e carga social’ assemelha-se àquele resultante de estudo que avaliou as propriedades psicométricas do Diabetes-39 entre pessoas com diabetes na Jordânia (32,7).^22^ Pessoas com diabetes sofrem mudanças no estilo de vida, dada a necessidade de seguir o tratamento, e essa alteração de hábito pode gerar limitações em atividades de lazer e no convívio com familiares e amigos.^28^ Sugere-se, portanto, que pessoas com diabetes estariam mais predispostas a desenvolver doenças como ansiedade e depressão, devido ao impacto social que a doença provoca.^29^

A avaliação da validade concorrente sugere que a escolaridade tem impacto positivo na qualidade de vida das pessoas com diabetes. Este resultado corrobora os de um estudo realizado na cidade de Ourense, Espanha, no ano de 2015, quando se sugeriu que a escolaridade e a compreensão da condição de saúde podem viabilizar práticas saudáveis, cuja aplicação no cotidiano das pessoas com diabetes contribui para a melhora da qualidade de vida.^30^

A avaliação da confiabilidade do Diabetes-21 apresentou consistência interna satisfatória, assim como o instrumento original, Diabetes-39.^3^^,^^22^ Os níveis de avaliação do Diabetes-21 viabilizaram a interpretação do construto ‘qualidade de vida entre pessoas com diabetes’. O mesmo estudo realizado na Jordânia, entre 368 pessoas com diabetes,^22^ avaliou a qualidade de vida utilizando o instrumento Diabetes-39 e apresentou forma semelhante de interpretação do instrumento adotada no presente trabalho, em que os valores foram transformados em uma escala de 0 a 100.

A redução dos itens do instrumento poderá gerar perda de comparabilidade, uma vez que a maioria dos estudos existentes utiliza o Diabetes-39. Nesse sentido, mais estudos psicométricos utilizando o Diabetes-21 devem ser realizados, para melhor se compreender sua estrutura e, afinal, considerá-lo válido e confiável.

O instrumento Diabetes-21 mostra-se promissor para avaliar a qualidade de vida relacionada à saúde entre pessoas com diabetes, e pode ser uma ferramenta útil para pesquisadores. Além disso, ele se apresenta como um instrumento em potencial para ser utilizado por profissionais de saúde no âmbito da assistência às pessoas com diabetes *mellitus*, por ser capaz de identificar comprometimento na qualidade de vida e possibilitar a implantação de ações que busquem minimizar os impactos da doença.

## Material Suplementar 1

**Table:** Versão final do instrumento Diabetes-21

Diabetes-21					
A qualidade de vida das pessoas é afetada por muitas coisas. Estas coisas podem incluir saúde, oportunidade de lazer e férias, amigos e família, e um trabalho. Este questionário é realizado para nos ajudar a compreender o que afeta a qualidade de vida de pessoas com diabetes. A seguir, pergunta-se sobre sua qualidade de vida. Para cada frase, assinale o grau em que concorda com as afirmativas, de acordo com a legenda: (1) não foi afetada (2) pouco afetada (3) às vezes afetada (4) muito afetada (5) extremamente afetada					
Durante o MÊS PASSADO, quanto sua qualidade de vida foi afetada:	1	2	3	4	5
1. Pela sensação de fraqueza					
2. Pelo quanto você consegue andar					
3. Pela necessidade de realizar exercícios regularmente					
4. Por não ser capaz de fazer atividades domésticas ou outros trabalhos que estão relacionados com a casa					
5. Pela necessidade de descansar várias vezes no dia					
6. Por dificuldades em cuidar de você mesmo(a) (de se vestir, tomar banho ou usar o vaso sanitário)					
7. Por andar mais devagar que os outros					
8. Pelas restrições alimentares necessárias para o controle do seu diabetes					
9. Por perder o controle dos níveis de açúcar no sangue					
10. Por ter que testar seus níveis de açúcar					
11. Por tentar manter seu diabetes bem controlado					
12. Pela necessidade de comer a intervalos regulares					
13. Pelas restrições que seu diabetes impõe sobre sua família e amigos					
14. Pelo constrangimento por ter diabetes					
15. Por fazer coisas que sua família ou seus amigos não fazem					
16. Pelo diabetes interferir na sua vida sexual					
17. Por problemas com função sexual					
18. Pela diminuição do interesse pelo sexo					
19. Pela preocupação relacionada com questões financeiras					
20. Pelo estresse ou pressão em sua vida					
21. Por sentimento de tristeza ou depressão					
**Avaliações gerais**					
O quanto o(a) senhor(a) está satisfeito(a) com sua qualidade de vida geral?					
(1) muito insatisfeito (2) insatisfeito (3) nem insatisfeito, nem satisfeito (4) satisfeito (5) muito satisfeito					
O quão grave você acha que é o seu diabetes?					
(1) nada grave (2) grave (3) mais ou menos grave (4) muito grave (5) extremamente grave					

## References

[1] Rodrigues AMAM, Cavalcanti AL, Pereira JLSH, Araújo CLC, Bernardino IM, Soares RL (2020). Use of the health services according to social determinants, health behaviors and quality of life among diabetics. Cien Saude Colet.

[2] Boyer JG, Earp JA (1997). The development of an instrument for assessing the quality of life of people with diabetes Diabetes-39. Med Care.

[3] Queiroz FA, Pace AE, Santos CB (2009). Cross-cultural adaptation and validation of the instrument Diabetes - 39 (D-39): brazilian version for type 2 diabetes mellitus patients - stage 1. Rev Lat Am Enfermagem.

[4] Miyamoto ST, Paganotti MA, Serrano EV, Giovelli RA, Valim V (2015). Assessment of fatigue and dryness in primary Sjögren's syndrome: Brazilian version of "Profile of Fatigue and Discomfort - Sicca Symptoms Inventory (short form) (PROFAD-SSI-SF)". Rev Bras Reumatol.

[5] Roque H, Veloso A, Ferreira PL (2016). Versão portuguesa do questionário EUROPEP: contributos para a validação psicométrica. Rev Saude Publica.

[6] Valim MD, Marziale MHP, Hayashida M, Rocha FLR, Santos JLF (2015). Validity and reliability of the Questionnaire for Compliance with Standard Precaution. Rev Saude Publica.

[7] Marques SRL, Lemos SMA (2017). Health literacy assessment instruments: literature review. Audiol Commun Res.

[8] Colucci MZO, Costa Alexandre NM, Milani D (2015). Construction of measurement instruments in the area of health. Cien Saude Colet.

[9] Mokkink LB, Terwee CB, Patrick DL, Alonso J, Stratford PW, Knol DL (2010). The COSMIN study reached international consensus on taxonomy, terminology, and definitions of measurement properties for health-related patient-reported outcomes. J Clin Epidemiol.

[10] Faria HTG, Veras VS, Xavier ATF, Teixeira CRS, Zanetti ML, Santos MA (2013). Quality of life in patients with diabetes mellitus before and after their participation in an educational program. Rev Esc Enferm USP.

[11] Instituto Brasileiro de Geografia e Estatística Cidades e estados: Montes Claros.

[12] Bertolucci PHF, Brucki SMD, Campacci SR, Juliano Y (1994). O mini-exame do estado mental em uma população geral: impacto da escolaridade. Arq Neuro-Psiquiatr.

[13] Hair JF, Black WC, Babin B, Anderson RE, Tatham RL (2009). Análise multivariada de dados.

[14] Luiz RR, Magnanini MMF (2000). A lógica da determinação do tamanho da amostra em investigações epidemiológicas. Cad. Saúde Coletiva.

[15] The World Health Organization (1995). Quality of Life Assessment (WHOQOL): position paper from the World Health Organization. Soc Sci Med.

[16] Landis JR, Koch GG (1977). The measurement of observer agreement for categorical data. Biometrics.

[17] Artes R (1998). Aspectos estatísticos da análise fatorial de escalas de avaliação. Rev Psiquiatr Clin.

[18] Reis E (1997). Estatística multivariada aplicada.

[19] Maroco JP (2014). Análise de equações estruturais: fundamentos teóricos, software & aplicações.

[20] Sousa LMM, Marques-Vieira CMA, Carvalho MLR, Veludo F, José HMG (2015). Fidelidade e validade na construção e adequação de instrumentos de medida. Enformação.

[21] Harper A, Power M (1996). Steps for checking and cleaning data and computing domain scores for the WHOQOL-bref.

[22] Khader YS, Bataineh S, Batayha W (2008). The Arabic version of Diabetes-39: psychometric properties and validation. Chronic Illn.

[23] Zulian LR, Santos MA, Veras VS, Rodrigues FFL, Arrelias CCA, Zanetti ML (2013). Quality of life in patients with diabetes using the diabetes 39 (D-39) instrument. Rev Gaucha Enferm.

[24] Lamu AN, Chen G, Gamst-Klaussen T, Olsen JA (2018). Do country-specific preference weights matter in the choice of mapping algorithms? The case of mapping the Diabetes-39 onto eight country-specific EQ-5D-5L value sets. Qual Life Res.

[25] Mahgoub AO, Abdelgadir E (2017). The association between health-related quality of life and Ramadan fasting in diabetic patients: a survey using a structured D-39 assessment tool. a Sudanese cohort. J Fasting Health.

[26] Rutherford D, Collier A (2005). Sexual dysfunction in women with diabetes mellitus. Gynecol Endocrinol.

[26] Enzlin P, Mathieu C, van Der Bruel A, Vanderschueren D, Demyttenaere K (2003). Prevalence and predictors of sexual dysfunction in patients with type 1 diabetes. Diabetes Care.

[28] Weinger K, Lee J (2006). Psychosocial and psychiatric challenges of diabetes mellitus. Nurs Clin North Am.

[29] Antúnez M, Bettiol AA (2016). Depression in patients with type 2 diabetes who attend an outpatient clinic of internal medicine. Acta Med Colomb.

[30] Fernández-Silva MJ, Alonso-González A, González-Pérez E, Gestal-Otero JJ, Díaz-Grávalos GJ (2019). Health literacy in patients with type 2 diabetes: a cross-sectional study using the HLS-EU-Q47 questionnaire. Semergen.

