# Supplementary material for: Validation of a reduced instrument Diabetes-21 for assessing health-related quality of life among people with diabetes
Source: Epidemiol Serv Saude. 2022 Mar 14;31(1):e2021324. [Article in Portuguese] doi: 10.1590/S1679-49742022000100004 (PMC11473133; doi:10.1590/S1679-49742022000100004)
Supplement: Translation

# Translation: Validation of a reduced instrument Diabetes-21 for assessing health-related quality of life among people with diabetes

**Authors:** Árlen Almeida Duarte de Sousa, Ana Monique Gomes Brito, Marise Fagundes Silveira, Andréa Maria Eleutério de Barros Lima Martins

**Affiliations:** 1 Universidade Estadual de Montes Claros, Departamento de Métodos e Técnicas Educacionais, Montes Claros, MG, Brazil Universidade Estadual de Montes Claros Universidade Estadual de Montes Claros Departamento de Métodos e Técnicas Educacionais Montes Claros MG Brazil; 2 Faculdades Unidas do Norte de Minas, Departamento de Enfermagem, Montes Claros, MG, Brazil Faculdades Unidas do Norte de Minas Faculdades Unidas do Norte de Minas Departamento de Enfermagem Montes Claros MG Brazil; 3 Universidade Estadual de Montes Claros, Programa de Pós-Graduação em Ciências da Saúde, Montes Claros, MG, Brazil Universidade Estadual de Montes Claros Universidade Estadual de Montes Claros Programa de Pós-Graduação em Ciências da Saúde Montes Claros MG Brazil

**Keywords:** Diabetes *Mellitus*, Quality of Life, Reproducibility of Results, Factor Analysis, Statistical

## Abstract

**Objective:**

To analyze the validity, reliability and interpretability of a short form instrument for assessing health-related quality of life among people with diabetes mellitus.

**Methods:**

This was a validation study, comprised of the adaptation phases of the Diabetes-39 instrument (consisting of 5 domains and 39 items), pre-test, structural validity analyses (exploratory and confirmatory), reliability, concurrent validity and interpretability.

**Results:**

The factorial structure of the short final version differed from the original instrument. The items were reduced from 39 to 21 and domains from 5 to 4. The factor loading, in exploratory and confirmatory analyses, ranged between 0.41 and 0.90 and between 0.51 and 0.89, respectively. Reliability was adequate (Cronbach’s alpha=0.91; Kappa≥0.60 in all items; intraclass correlation coefficient =0.91).

**Conclusion:**

Diabetes-21, a short form instrument, was considered valid, reliable and interpretable for assessing health-related quality of life among people with diabetes *mellitus*.

**Study contributions**

**Main results**

Diabetes-21 was considered to be valid, reliable and interpretable for assessing health-related quality of life among people with diabetes attending primary care services.

**Implications for services**

It is an instrument that has potential for use by health professionals in providing care for people with diabetes, since it is capable of identifying impaired quality of life, as well as enabling health actions to be implanted.

**Perspectives**

It is expected that Diabetes-21 will be used in future research and also by health services to identify people in need of intervention, especially since it requires less application time.

## Introduction

Diabetes *mellitus* is one of the leading causes of premature and preventable deaths in Brazil. The quality of life of the population frequently affected by health problems associated with diabetes reinforces the need to evaluate this condition, in addition to the follow-up and clinical treatment of the disease.^1^

The quality of life of people with diabetes *mellitus* has been assessed by the Diabetes-39 instrument, developed in the United States and adapted for Brazil. It is a multidimensional scale, comprised of 39 items that assess five domains of the quality of life of people with diabetes: energy and mobility; diabetes control; anxiety and worry; social burden; and sexual functioning. The higher the score obtained on the scale, the greater the negative impact on people’s quality of life.^2^^,^^3^

Diabetes-39 has been considered an adequate instrument to assess the quality of life of this population. However, in its cross-cultural adaptation for the Brazilian population, internal consistency and construct validity (convergent and discriminant validity) were the only psychometric properties assessed, therefore it is necessary to analyze its stability, structural validity (exploratory and confirmatory factor analyses) and interpretability.^3^ Although the Brazilian version has maintained the validity and reliability of the original version, it is a long instrument that requires considerable time and adequate environment for its application. The limitations of the instrument may discourage or create barriers to participation, both for people with diabetes and health professionals, while short questionnaires are rapidly applied, practical and economical.^4^^-^^6^

The validation of the efficacy of instruments that assess health conditions is crucial, as they may present limitations in their psychometric properties.^7^^-^^9^ A group of reseachers who are experts in the evaluation of measurement instruments developed the ‘COnsensus-based Standards for the selection of health Measurement INstruments’ (COSMIN), a checklist for analyzing the psychometric properties of instruments that assess health conditions. COSMIN, present in four domains, evaluates the validity, reliability, responsiveness and interpretability of the instruments.^8^^,^^9^ Epidemiological studies using instruments that have already been tested, regarding their validity and reliability, contribute to evidence-based practices in healthcare.^10^

Diabetes-39, when assessing the health-related quality of life of people with diabetes, contributes to identifying care needs and, consequently, reducing the risk of complications arising from the disease. A short version of this instrument may contribute to a shorter time of its application in epidemiological studies. In addition, during the validation process of an instrument, factor loadings or weights are obtained, which enable the estimation of a score in line with the importance that each question/item exerts on the construct. Therefore, considering the weight of each question/item in the interpretation of the instrument can lead to a more reliable result.^9^

This study aimed to analyze the validity, reliability and interpretability of a short form instrument for assessing health-related quality of life among people with diabetes *mellitus*.

## Methods

### Study design

This was a cross-sectional validation study conducted between 2016 and 2018, with users of Family Health Strategy (FHS) units in the city of Montes Claros, state of Minas Gerais, Brazil.

### Context

Montes Claros, located in the north of Minas Gerais, is the sixth largest city in the state by population, occupies an area of 3,589,811km^2^ and had a human development index (HDI) of 0.770 in 2010. In 2020, its population was estimated at 413,487 inhabitants.^11^

### Population and sample

The municipal management responsible for the FHS was contacted in order for us to obtain the lists with the enumeration of the family health strategy teams centers. Of the 73 health centers in Montes Claros, four were selected using simple random draw. Of these, two were used to assess the reliability and concurrent validity, and the other two health centers were used to estimate the structural validity and interpretability.

The managers of the FHS centers that had been randomly selected provided lists with the names of people with diabetes who were registered and assisted by FHS teams. Based on these data, people aged 18 years or older, registered in the FHS and who had diabetes, were considered eligible for this study. Those with three or more comorbidities, whose native language was not Portuguese, who had visual or hearing impairment and presented signs of alcohol or drug intoxication at the time of the interviews; and the elderly with cognitive impairment, which could be verified using the mini mental state examination, were excluded.^12^ The instruments that presented three or more incomplete data were considered losses.

Thus, the study sample was divided into two different groups:

1. The reliability group (internal consistency and stability) and concurrent validity of the instrument, in which 50 people with diabetes registered in the health units were selected, given that the samples consisting of 50 to 100 people were sufficient for these stages;^13^ and
2. The group of exploratory and confirmatory factor analyses, and interpretability, in which the participants were selected through the calculation of a probabilistic sampling for an infinite population, taking into consideration the proportion of 50% of people with diabetes and quality of life impairment, 95% confidence level, sampling error of 6 percentage points and an increase of 10% for losses.^14^

### Variables

The sociodemographic and economic status investigated were: sex (male; female); age group (in years, categorized into quartiles: 22 to 54; 55 to 61; 62 to 68; 69 or over); schooling (in complete years of study: 0; 1 to 4; 5 to 8; 9 to 11; 12 or more); marital status (married/stable union; single/widowed/divorced/ separated); race/skin color (White; Asian; Black; Brown; Indigenous; had no information); family income [up to BRL 998.00; above BRL 998.00 (minimum wage at the time of the study)]; and spending on medicines (no; yes).

Cognitive impairment, exclusion criteria of the study, was defined according to different cut-off points - based on schooling - using the mini mental state examination: 13 for people without education; 18 for low and medium schooling levels (1 to 8 incomplete years of schooling); and 26 for high schooling level (8 or more years of schooling).^12^

Participants whose scores fell below the lower limit of 95% confidence interval (95%CI) for the mean in each dimension were classified as having impaired quality of life (yes; no).

### Data sources and measurement

Diabetes-39, the instrument that was assessed, was culturally adapted to Brazilian Portuguese.^3^ Its 39 items are distributed into five domains: energy and mobility; diabetes control; social burden; sexual functioning; anxiety and worry. Its short version was called Diabetes-21.

In addition, a change in the response scale was made (1 = was not affected; 2 = little affected; 3 = sometimes affected; 4 = very affected; 5 = extremely affected), replacing the original proposal of Diabetes-39 (horizontal bar divided into boxes, that were numbered from 1 to 7).^3^

To verify the validity, reliability and interpretability of Diabetes-21, the following steps were applied: pre-test, structural validity (exploratory and confirmatory factor analysis), concurrent validity, reliability (internal consistency and stability) and interpretability.

For the pre-test, 20 individuals with diabetes who were selected using convenience sampling and registered in the first health unit selected to compose the sample for the study, were interviewed. The objective of this stage was to analyze the applicability of the instrument before its use in the final sample of the study. After the pre-test, the interviewers were invited to hold a meeting with the researchers to report their perception regarding the application of the instrument with a new response scale. This meeting was conducted as a focus group, comprised of ten judges who worked as researchers and/or providing care to people with diabetes (2 endocrinologists, 2 epidemiologists, 2 nurses, 1 physiotherapist, 1 nutritionist, 1 physical education teacher and 1 dentist).

In addition to Diabetes-39, this study used the short form of World Health Organization Quality Of Life - BREF (WHOQOL-BREF). It is an instrument for assessing quality of life, but it is not specific for people with diabetes, consists of two general questions and 26 related to four domains: physical, psychological, environmental and social. Their answers consider a Likert scale that measures intensity, frequency, capacity and evaluation. The scores of each domain were transformed into a scale from 0 to 100, reversed and dichotomized by the lower limit of 95%CI for the mean. Individuals whose scores fell below this limit, in each domain, were classified as having impaired quality of life.^15^

### Bias control

The participants - from all stages of the study - were interviewed at home, individually and in a reserved room.

The measurement bias was minimized through the theoretical and practical training of the interviewers. They were trained to minimize the intrinsic subjectivity of the interviews. The training included people with diabetes who did not take part in the study.

### Statistical analyses

Descriptive analysis of categorical variables was performed, estimating absolute and relative frequencies. For the continuous variables, the mean and standard deviation (SD) were calculated, and the 95%CI was estimated, in addition to minimum and maximum values.

To estimate concurrent validity, Spearman’s correlation coefficient was used to verify the association between the variables of the instruments (WHOQOL-BREF and Diabetes-21; Diabetes-39 and Diabetes-21), given that the data did not present normal distribution. Then, the correlation test was applied between the scales generated by these same instruments. The correlations between Diabetes-21 scores and the age and schooling of the participants were also analyzed.

The reliability of the instrument was measured through internal consistency and stability. Internal consistency was tested using Cronbach’s alpha calculation (α), and values of ≥0.7 were considered acceptable.^13^ To estimate the stability of the instrument, the test-retest was applied, in which the ability of the instrument to produce similar results was verified, measuring the event among the same participants in different situations, by calculating the intraclass correlation coefficient. The test-retest was applied with an interval of 7 to 14 days.^5^^,^^13^ The weighted Kappa coefficient was calculated to assess the agreement of each of the items of the short version of the instrument, considering as acceptable the cutoff point ≥0.60.^16^

The evaluation of the structural validity of Diabetes-39 was performed using exploratory and confirmatory factor analyses:

- Regarding exploratory factor analysis, the correlation matrix of the instrument variables was analyzed (considered significant p-value<0.05), along with the following tests and analyses:^17^^-^^19^ (i) the Kaiser-Meyer-Olkin test (ranging from 0 to 1; values <0.50 indicate inadequate method);^18^ (ii) Bartlett’s scouting test (p-value<0.05 indicates that the correlation matrix differs from an identity matrix and therefore there are relationships among the variables included in the analysis); (iii) the identification of commonalities (estimation of the shared or common variance between variables) and their contribution to each item (>0.5 acceptable levels of explanation; lower or equal values indicate that the item should be excluded); (iv) the analysis of the factor loadings of each item regarding the extracted components (0.40 as an acceptable limit of the contribution of the item to the creation of the factor); (v) the definition of the number of factors, based on the graphical evaluation of the scree plot (graph of eigenvalues versus number of factors in the order of extraction), in the verification of the eigenvalue (greater than 1) and observation of the cumulative percentage of the total variance; and (vi) principal component analysis, in which the variables were rotated - orthogonal rotation (varimax).
- With the regard to confirmatory factor analysis, the maximum likelihood method was used.^18^ To adjust the model, the following indices were considered:^19^^,^^20^ (i) the ratio between Pearson’s chi-square and the degrees of freedom (excellent = 1-2; good = 2-3; acceptable = 4-5; rejected = >5); (ii) the quality index of the adjustment (adequate values: ≥0.90); (iii) the root mean square error of approximation (adequate <0.08); (iv) the comparative fit index (adequate values: ≥0.90); and (v) the Tucker-Lewis index (adequate values: ≥0.90).

The order of the questions was reorganized in the short form instrument. To interpret it, we used the weighted additive model, taking into consideration the factor loading attributed to each item, for example: Question 9 x 0.63 (factor loading).^13^ These estimates were performed through the ratio between the sum of different items that constitute the factors, multiplied by the respective factorial weights (factor loading), and the sum of the factorial weights that were attributed, being considered the best quality of life that one that achieved the lowest score, given that it is a Likert-type response scale, from 1 (not affected) to 5 (extremely affected). The variable was transformed into a binary categorical variable when a cutoff point was considered; as such, the values were transformed into a scale from 0 to 100 ^14^^,^^21^^,^^22^ reversed and dichotomized by the lower limit of 95%CI for the mean. Thus, participating individuals who scored below this limit in each domain (energy and mobility, 35.91; diabetes control and social burden, 32.49; sexual functioning, 31.47; anxiety and worry, 38.10) were considered with impaired quality of life.^22^

Statistical Package for the Social Science (SPSS) version 24.0 and Microsoft Excel were used for all analyses.

### Ethical aspects

The study was approved by the Research Ethics Committee of the Universidade Estadual de Montes Claros (CEP/Unimontes) on March 22, 2016: Opinion No. 1,461,818; Certificate of Submission for Ethical Appreciation (CAAE) No. 54417616.1.0000.5146. All participants signed the Free and Informed Consent Form.

## Results

The mean age of the 50 participants of the verification stage of reliability and concurrent validity was 61 years (SD=11.1; minimum value, 36; maximum value, 87) and mean years of schooling of 7.5 (SD=3.9; minimum value, 0; maximum value, 12). At this stage, there were no exclusions, losses or refusals of the participants.

A total of 297 participants were invited to structural analysis and interpretability stage (Figure 1). However, due to 5 losses and 4 refusals, 288 people with diabetes, whose mean age was 60 years (ranging from 22 to 92 years: SD=11.7) took part in the study. The sociodemographic, economic and quality of life information of the participants can be found in Table 1.

**Figure 1:** Selection process of people with diabetes *mellitus* assisted by the Family Health Strategy, Montes Claros, state of Minas Gerais, Brazil, 2019

**Table 1:** Distribution of sociodemographic and economic characteristics and quality of life among people with diabetes *mellitus* (n=338) assisted by the Family Health Strategy, Montes Claros, state of Minas Gerais, Brazil, 2019

Sociodemographic and economic status	Study Population			Quality of life affected in the Diabetes-21 domains (n=288)															
	n=50	n=288		Sexual functioning				Energy e mobility				Diabetes control and social burden				Anxiety and worry			
	Total			Yes		No		Yes		No		Yes		No		Yes		No	
	N	N	%	N	%	N	%	N	%	N	%	N	%	N	%	N	%	N	%
**Sex**																			
Female	34	189	65.6	133	73.1	56	52.8	93	59.2	96	73.3	100	64.1	89	67.4	87	60.0	102	71.3
Male	16	99	34.4	49	26.9	50	47.2	64	40.8	35	26.7	56	35.9	43	32.6	58	40.0	41	28.7
**Age group (years)^a^**																			
22-54	8	81	28.1	50	27.5	31	29.3	39	24.8	42	32.1	37	23.7	44	33.4	29	20.0	52	36.3
55-61	14	62	21.5	39	21.4	23	21.7	35	22.3	27	20.6	30	19.2	32	24.2	28	19.3	34	23.8
62-68	11	78	27.1	43	23.6	35	33.0	45	28.7	33	25.2	46	29.5	32	24.2	45	31.0	33	23.1
≥69	11	67	23.3	50	27.5	17	16.0	38	24.2	29	22.1	43	27.6	24	18.2	43	29.7	24	16.8
**Schooling (years)^a^**																			
0	2	17	6.0	12	6.7	5	4.7	8	5.3	9	7.0	8	5.1	9	6.8	8	5.5	9	6.2
1-4	11	65	22.6	37	20.3	28	26.7	28	17.9	37	28.2	34	21.9	31	23.5	30	20.8	35	24.5
5-8	11	89	31.0	59	32.4	30	28.6	52	33.3	37	28.2	48	31.0	41	31.1	43	29.9	46	32.2
9-11	10	49	17.1	31	17.0	18	17.1	28	17.9	21	16.0	28	18.1	21	15.9	25	17.4	24	16.8
≥12	8	67	23.3	43	23.6	24	22.9	40	25.6	27	20.6	37	23.9	30	22.7	38	26.4	29	20.3
**Marital statu**s																			
Married/stable union	33	174	60.4	103	56.5	71	67.0	96	61.1	78	59.5	88	56.4	86	65.2	85	58.6	89	62.2
Single/widowed/divorced/separated	17	114	39.6	79	43.5	35	33.0	61	38.9	53	40.5	68	43.6	46	34.8	60	41.4	54	37.8
**Race/skin color**																			
White	22	89	30.9	58	31.9	31	29.2	47	29.9	42	32.1	46	29.5	43	32.5	46	31.7	43	30.0
Asian	4	14	4.9	7	3.8	7	6.6	5	3.2	9	6.9	5	3.2	9	6.8	5	3.4	9	6.3
Black	4	36	12.5	25	13.7	11	10.4	20	12.7	16	12.2	16	10.3	20	15.2	14	9.7	22	15.4
Brown	20	134	46.5	82	45.1	52	49.1	75	47.8	59	45.0	79	50.6	55	41.7	72	49.7	62	43.4
Indigenous	-	3	1.0	2	1.1	1	0.9	3	1.9	-	0.0	3	1.9	-	0.0	2	1.4	1	0.7
No information provided	-	12	4.2	8	4.4	4	3.8	7	4.5	5	3.8	7	4.5	5	3.8	6	4.1	6	4.2
**Household income^a^**																			
Up to BRL 998.00	8	81	30.2	54	31.8	27	27.6	38	26.2	43	35.0	38	26.2	43	35.0	34	25.6	47	34.8
Over BRL 998.00	42	187	69.8	116	68.2	71	72.4	107	73.8	80	65.0	107	73.8	80	65.0	99	74.4	88	65.2
**Spending on medicines**																			
Yes	-	147	51.0	89	48.9	52	49.1	79	50.3	62	47.3	78	50.0	63	47.7	74	51.0	67	46.9
No	-	141	49.0	93	51.1	54	50.9	78	49.7	69	52.7	78	50.0	69	52.3	71	49.0	76	53.1

a) The number of respondents is lower than the number of participants, due to refusal to answer this variable.

The internal consistency of Diabetes-21 was high (α=0.91). Furthermore, the removal of any of the items did not alter the internal consistency of its construct (all items presented α=0.91). The agreement of the 21 items of the instrument was adequate (Kappa ≥0.60) (Table 2). Stability was good (intraclass correlation coefficient=0.91).

**Table 2:** Domain agreement, according to the removal of each item of Diabetes-21 (n=50), among people with diabetes *mellitus* assisted by the Family Health Strategy, Montes Claros, state of Minas Gerais, Brazil, 2019

Item and domains	Weighted Kappa	p-value^a^
**Energy and mobility**		
1. By feeling of weakness	0.67	<0.001
2. By how far you can walk	0.60	<0.001
3. By any daily exercise requirements	0.85	<0.001
4. By not being able to do housework or other jobs around the house	0.93	<0.001
5. By needing to rest often	0.83	<0.001
6. By having trouble caring for yourself (dressing, bathing or using the toilet)	0.76	<0.001
7. By walking more slowly than others	0.92	<0.001
**Diabetes control and social burden**		
8. By food restrictions required to control your diabetes	0.82	<0.001
9. By losing control of your blood sugar levels	0.76	<0.001
10. By testing your blood sugar levels	0.77	<0.001
11. By getting your diabetes well controlled	0.82	<0.001
12. By the need to eat at regular intervals	0.83	<0.001
13. By the restrictions your diabetes places on your family and friends	0.84	<0.001
14. By being embarrassed because you have diabetes	0.73	<0.001
15. By doing things your family and friends do not do	0.87	<0.001
**Sexual functioning**		
16. By diabetes interfering with your sex life	0.61	<0.001
17. By problems with sexual functioning	0.90	<0.001
18. By a decreased interest in sex	0.87	<0.001
**Anxiety and worry**		
19. By worries about money matters	0.82	<0.001
20. By stress or pressure in your life	0.90	<0.001
21. By feeling depressed or low	0.90	<0.001

a) Z-test, Weighted Kappa.

The factorial structure of the final version of Diabetes-21 showed differences regarding the original structure. Questions 1, 3, 4, 6, 7, 12, 13, 14, 16, 18, 25, 27, 31, 33, 35, 37, 38 and 39 from Diabetes-39 instrument were removed, because they showed commonality lower than 0.5. Thus, it was reduced, from 39 to 21 items, with a decrease in the number of domains from 5 to 4, observed in exploratory factor analysis (energy and mobility; diabetes control and social burden; sexual functioning; anxiety and concern), due to the union of the dimensions ‘diabetes control’ and ‘social burden’. The union of these two dimensions resulted in better internal consistency and stability of the instrument. All dimensions presented good internal consistency (α≥0.70). Factor loadings ranged from 0.41 (item 15) to 0.90 (item 17); the dimension ‘sexual functioning’ presented the highest factor loading (values >0.75) (Table 3).

**Table 3:** Commonalities, factor loadings of each item by extracted factor and Cronbach’s alpha (α) by Diabetes-21 factor (n=288),^a^ among people with diabetes *mellitus* assisted by the Family Health Strategy, Montes Claros, state of Minas Gerais, Brazil, 2019

Diabetes-21 - Item and domains	Commonalities	Factor 1	Factor 2	Factor 3	Factor 4	Cronbach’s alpha
Factor: energy and mobility						
1. By feeling of weakness	0.47	0.49	0.20	0.16	0.41	0.83
2. By how far you can walk	0.62	0.72	0.26	0.03	0.19	
3. By any daily exercise requirements	0.46	0.59	0.34	-0.01	0.01	
4. By not being able to do housework or other jobs around the house	0.61	0.74	0.07	0.21	0.10	
5. By needing to rest often	0.57	0.70	0.18	0.17	0.17	
6. By having trouble caring for yourself (dressing, bathing or using the toilet)	0.43	0.61	0.05	0.20	0.11	
7. By walking more slowly than others	0.61	0.67	0.298	0.12	0.25	
**Factor: diabetes control and social burden**						
8. By food restrictions required to control your diabetes	0.55	0.06	0.73	-0.07	0.11	0.86
9. By losing control of your blood sugar levels	0.46	0.28	0.54	0.22	0.20	
10. By testing your blood sugar levels	0.60	0.24	0.71	0.06	0.17	
11. By getting your diabetes well controlled	0.63	0.17	0.73	0.23	0.13	
12. By the need to eat at regular intervals	0.58	0.28	0.66	0.23	0.13	
13. By the restrictions your diabetes places on your family and friends	0.62	0.33	0.64	0.23	0.22	
14. By being embarrassed because you have diabetes	0.49	0.14	0.47	0.29	0.40	
15. By doing things your family and friends do not do	0.54	0.57	0.41	0.10	0.19	
**Factor: sexual functioning**						
16. By diabetes interfering with your sex life	0.79	0.16	0.14	0.85	0.10	0.86
17. By problems with sexual functioning	0.84	0.09	0.14	0.90	0.07	
18. By a decreased interest in sex	0.71	0.29	0.20	0.76	0.11	
**Factor: anxiety and worry**						
19. By worries about money matters	0.56	0.15	0.16	0.07	0.71	0.70
20. By stress or pressure in your life	0.77	0.16	0.19	-0.00	0.84	
21. By feeling depressed or low	0.55	0.32	0.22	0.23	0.59	

a) Analysis performed from factor extraction using the principal component method, followed by a orthogonal rotation (varimax).

The eigenvalues found in each factor were: 1.48 (energy and mobility, factor 1); 1.18 (diabetes control and social load, factor 2); 8.00 (sexual functioning, factor 3); and 1.77 (anxiety and worry, factor 4). The variance explained by each factor was 7.1% (factor 1), 5.6% (factor 2), 38.1% (factor 3) and 8.5% (factor 4).

The results of the Kaiser-Meyer-Olkin test (0.91) and Bartlett’s test of sphericity (2,579.51) of Diabetes-21 were satisfactory and adequate (p<0.001). Their structure was explained by four latent factors, indicated by the graphical evaluation of the scree plot (inflection point of the curve). The factors explained 59.3% of the total variance. The final version of Diabetes-21 is presented as a supplementary material (Supplementary Material 1).

Confirmatory factor analysis showed indices adequate to the tested model (ratio between chi-square and degrees of freedom, 2.22; fit quality index, 0.88; the root mean square error of approximation, 0.06; comparative fit index, 0.91; Tucker-Lewis index, 0.90). Factor loadings ranged from 0.51 (Q34) to 0.89 (Q23). Figure 2 systematizes confirmatory factor analysis of Diabetes-21.

**Figure 2:** Confirmatory factor analysis of Diabetes-21 (n=288) among people with diabetes *mellitus* assisted by the Family Health Strategy, Montes Claros, state of Minas Gerais, Brazil, 2019

There was a negative correlation with age (Spearman’s correlation coefficient = -0.14; p=0.021) and positive correlation with schooling (Spearman’s correlation coefficient = 0.76; p=0.001). Spearman’s correlation coefficient value between the total score of Diabetes-39 and Diabetes-21 was strong (0.97; p<0.001). It could be seen a mean correlation between the total score of the WHOQOL-BREF and the Diabetes-21 (0.51; p<0.001).

The mean values found in the Diabetes-21 domains were: energy and mobility (37.85; SD=16.71; 95%CI 35.91;39.78); diabetes control and social burden (34.22; SD=14.93; 95%CI 32.4 9;35.96); sexual functioning (33.77; SD=19.80; 95%CI 31.47;36.06); anxiety and worry (40.03; SD=16.60; 95%CI 38.10;41.95).

Poor health-related quality of life was more frequent in the domain ‘sexual functioning’ (63.2%), followed by the domains ‘energy and mobility’ (54.5%; n=157), ‘diabetes control and social burden’ (54.2%; n=156) and ‘anxiety and worry’ (50.3%; n=145).

## Discussion

Diabetes-21 instrument was considered to be valid, reliable and interpretable by the users of primary health care in Montes Claros, state of Minas Gerais. Regarding exploratory factor analysis, it could be seen an association between the items present in the domains ‘social burden’ and ‘diabetes control’, and the union of these domains was performed. The confirmatory factor analysis showed satisfactory adjustment for the acceptance of the proposed theoretical model. With regard to the concurrent validity of Diabetes-21, it could be seen that the highest level of quality of life was positively correlated with schooling; and negatively with age. Estimates of reliability, in general and within each domain, showed satisfactory reliability, both in stability and internal consistency. Diabetes-21 instrument was able to discriminate levels of quality of life among participants, showing good reliability and validity.

Some limitations of the study should be taken into consideration. Convenience sampling was used to verify reliability and concurrent validity; this type of sampling presents limitations regarding the results and conclusions, given that they cannot be generalized to the entire population, due to the selection bias. However, the results of this sample show a variability related to the variables investigated, suggesting that it may represent the general population. Criterion validity includes estimates of concurrent and predictive validity, although predictive validity could not be estimated due to the absence of a gold standard.^9^ Thus, the concurrent validity was estimated by the correlation between the scores obtained from Diabetes-21 related to the age and schooling of the participants. Responsiveness was not estimated because this is a cross-sectional study that was not designed to detect changes over time.

No structural validation studies on Diabetes-39 with reduction of items or domains were found. In this study, the reduction was based on the need to review the instruments before applying them in specific samples, because both intrinsic and extrinsic factors to the disease may change over time. The results found in this research corroborate those of previous studies, because when comparing Diabetes-39 with other instruments, it was considered to be adequate, as it had been previously used in the investigation of quality of life among people with diabetes.^23^^-^^25^

Regarding Diabetes-21, it could be seen that poor quality of life was more frequent in the domain ‘sexual functioning’. Several factors are related to sexual dysfunction among people with diabetes. Vascular anomalies, endocrine disorders, emotional impact and medications used for the treatment can cause ejaculation problems, erectile dysfunction and arousal disorder, and thus, decreased desire.^26^^,^^27^ As such, sexual functioning should be taken into consideration in the treatment of diabetes, in the same way as any emotional aspects that can impact people’s quality of life.^23^

The mean value showed in the domain ‘diabetes control and social burden’ is similar to that found in a study that evaluated the psychometric properties of Diabetes-39 among people with diabetes in Jordan (32,7).^22^ People with diabetes undergo lifestyle changes, given the need to follow treatment, and this behavior change can result in reduction in leisure activities and the time spend with family and friends.^28^ Therefore, it is believed that people with diabetes are more likely to develop diseases such as anxiety and depression, due to the social impact that this disease causes.^29^

The assessment of concurrent validity suggests that schooling has a positive impact on the quality of life of people with diabetes. This result corroborates those of a study conducted in the city of Ourense, Spain, in 2015, when it was suggested that schooling and the understanding of health condition can enable the adoption of healthy habits in the daily lives of people with diabetes, contributing to the improvement of quality of life.^30^

The assessment of Diabetes-21 reliability, as well as the original instrument, Diabetes-39, showed satisfactory internal consistency.^3^^,^^22^ The levels of assessment of Diabetes-21 enabled the interpretation of ‘quality of life construct among people with diabetes’. The same study conducted in Jordan, among 368 people with diabetes,^22^ assessed quality of life using the Diabetes-39 instrument and also a similar form of interpretation of the instrument adopted in this study, in which the values were transformed to a 0 to 100 scale.

The reduction of the instrument’s items may generate loss of comparability, given that most existing studies have used Diabetes-39. As such, more psychometric studies using Diabetes-21 should be carried out in order to better understand its structure and thus, consider it valid and reliable.

Diabetes-21 instrument proved to be promising for assessing health-related quality of life among people with diabetes, and can be a useful tool for researchers. In addition, it is as an effective instrument to be used by health professionals in the context of care for people with diabetes *mellitus*, because it is able to identify impairment in quality of life and enable the implementation of actions aimed at minimizing the impacts of the disease.

## Supplementary Material 1

**Table:** Final version of Diabetes-21

Diabetes-21					
People’s quality of life is affected by many things. These things might include health, leisure and vacation opportunities, friends and family, and a job. This questionnaire is designed to help us understand what affects the quality of life of people with diabetes. Below, there are some questions about your quality of life. For each sentence, put a check in the box to show how much that factor affects your quality of life, according to the legend: (1) not affected (2) little affected (3) sometimes affected (4) very affected (5) extremely affected					
During the past month, how much was the quality of your life affected by:	1	2	3	4	5
1. feelings of weakness					
2. how far you can walk					
3. Any daily exercise requirements					
4. not being able to do housework or other jobs around the house					
5. needing to rest often					
6. having trouble caring for yourself (dressing, bathing or using the toilet)					
7. walking more slowly than others					
8. food restrictions required to control your diabetes					
9. losing control of your blood sugar levels					
10. having to test your blood sugar levels					
11. getting your diabetes well controlled					
12. the need to eat at regular intervals					
13. the restrictions your diabetes places on your family and friends					
14. being embarrassed because you have diabetes					
15. doing things that your family and friends don’t do					
16. diabetes interfering with your sex life					
17. problems with sexual functioning					
18. a decreased interest in sex					
19. worries about money matters					
20. stress or pressure in your life					
21. feeling depressed or low					
**Overall assessment**					
How satisfied are you with your overall quality of life?					
(1) not satisfied at all (2) dissatisfied (3) neither satisfied nor dissatisfied (4) satisfied (5) very satisfied					
How serious do you think your diabetes is?					
(1) not serious (2) serious (3) more or less serious (4) very serious (5) extremely serious
